# Three-step operation for esophago-left bronchial fistula with respiratory failure after esophagectomy: a case report with literature review

**DOI:** 10.1186/s12876-021-02051-6

**Published:** 2021-12-14

**Authors:** Yuta Sato, Yoshihiro Tanaka, Tomonari Suetsugu, Ritsuki Takaha, Hidenori Ojio, Yuji Hatanaka, Takeharu Imai, Naoki Okumura, Nobuhisa Matsuhashi, Takao Takahashi, Hisakazu Kato, Kazuhiro Yoshida

**Affiliations:** 1 Department of Gastroenterological Surgery and Pediatric Surgery, Gifu University Graduate School of Medicine, Gifu City, Japan; 2 Department of Plastic and Reconstructive Surgery, Gifu University Graduate School of Medicine, Gifu City, Japan

**Keywords:** Esophageal cancer, Esophagectomy, Esophago-bronchial fistula, Esophagostomy, Three-step method

## Abstract

**Background:**

The development of esophago-bronchial fistula after esophagectomy and reconstruction using a posterior mediastinal gastric tube remains a rare complication associated with a high rate of mortality.

**Case presentation:**

A 63-year-old man with esophageal cancer underwent a thoracoscopic esophagectomy with two-field lymph node dissection and reconstruction via a gastric tube through the posterior mediastinal route. Postoperatively, the patient developed extensive pyothorax in the right lung due to port site bleeding and hematoma infection. Four months after surgery, he developed an esophago-left bronchial fistula due to ischemia of the cervical esophagus and severe reflux esophagitis at the site of the anastomosis. Because of respiratory failure due to the esophago-bronchial fistula and the history of extensive right pyothorax, right thoracotomy and left one-lung ventilation were thought to be impossible, so we decided to perform the surgery in three-step systematically. First, we inserted a decompression catheter and feeding tube into the gastric tube as a gastrostomy and expected neovascularization to develop from the wall of the gastric tube through the anastomosis after this procedure. Second, 14 months after esophagectomy, we constructed an esophagostomy after confirming blood flow in the distal side of the cervical esophagus via gastric tube using intraoperative indocyanine green-guided blood flow evaluation. In the final step, we closed the esophagostomy and performed a cervical esophago-jejunal anastomosis to restore esophageal continuity using a pedicle jejunum in a Roux-en-Y anastomosis via a subcutaneous route.

**Conclusion:**

This three-step operation can be an effective procedure for patients with esophago-left bronchial fistula after esophagectomy, especially those with respiratory failure and difficulty in undergoing right thoracotomy with left one-lung ventilation.

## Background

Esophago-bronchial fistula (EBF) is a rare but life-threatening complication after subtotal esophagectomy with gastric tube reconstruction via the posterior mediastinal route. Refractory EBF necessitates a surgical procedure, which generally requires a thoracotomy and one-lung ventilation to approach the fistula. However, such procedures might be too invasive for patients with pneumonia and respiratory failure caused by the EBF. We report a rare case of EBF after esophagectomy for esophageal cancer that was successfully treated by a three-step operation without thoracotomy. The first step aimed at improving pneumonia and nutritional status and expected neovascularization to develop from the wall of the gastric tube through the anastomosis, the second created an esophagostomy using intraoperative indocyanine green (ICG) to evaluate blood flow through the anastomosis, and the third restored esophageal continuity using an esophago-jejunal bypass.

## Case presentation

**Fig. 1:**
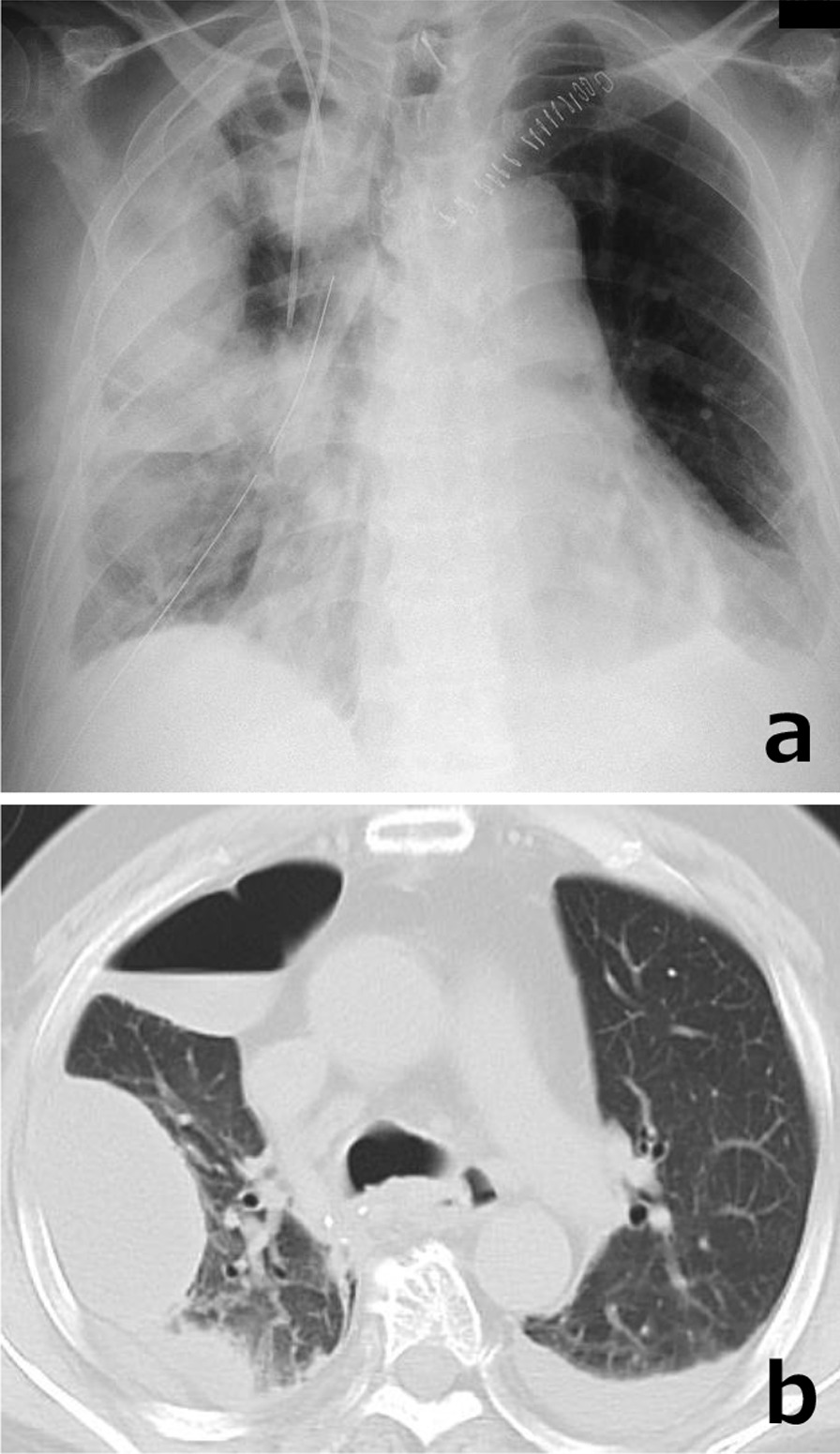
Chest X-ray and CT images on POD 7. **a** Lobar pneumonia of the right lung and pyothorax of the right middle and upper lobes. **b** Multiple pyothoraces, pneumonia and pneumothorax of the right lung and bilateral pleural effusion

A 63-year-old man was diagnosed as having advanced esophageal cancer by upper endoscopy after a chief complaint of dysphagia. The tumor was located in the lower thoracic esophagus and a biopsy revealed squamous cell carcinoma. His body weight was 98 kg and body mass index was 30.2 kg/m^2^. Preoperative diagnosis of the tumor was T3N1M0 Stage III according to the TNM classification of the Union for International Cancer Control [1]. After he received two courses of bi-weekly DCF (docetaxel 35 mg/m^2^, cisplatin 40 mg/m^2^, and fluorouracil 400 mg/m^2^) [2, 3] as neoadjuvant chemotherapy, we performed thoracoscopic esophagectomy with two-field lymphadenectomy, subtotal stomach reconstruction via the posterior mediastinal route, and jejunostomy. The cervical esophago-subtotal stomach anastomosis was created via the hand-sewn method. On postoperative day (POD) 7, the patient complained of high fever and cough, and chest X-ray and computed tomography (CT) revealed a right pyothorax and air leakage (Fig. 1a, b). Upper gastrointestinal imaging showed no anastomotic leakage. Also, multiple right thoracic cavity drainage was performed, but no turbulent fluid flow was obtained from the thoracic tube. Therefore, we considered that the extensive pyothorax and pneumothorax had developed due to port site bleeding and hematoma infection. After conservative therapy including drainage and antibiotics, the inflammation gradually improved. The patient resumed oral intake on POD 14 and was discharged from hospital on POD 31. He was admitted to another hospital with pneumonia once after esophagectomy and treated with antibiotics.

**Fig. 2:**
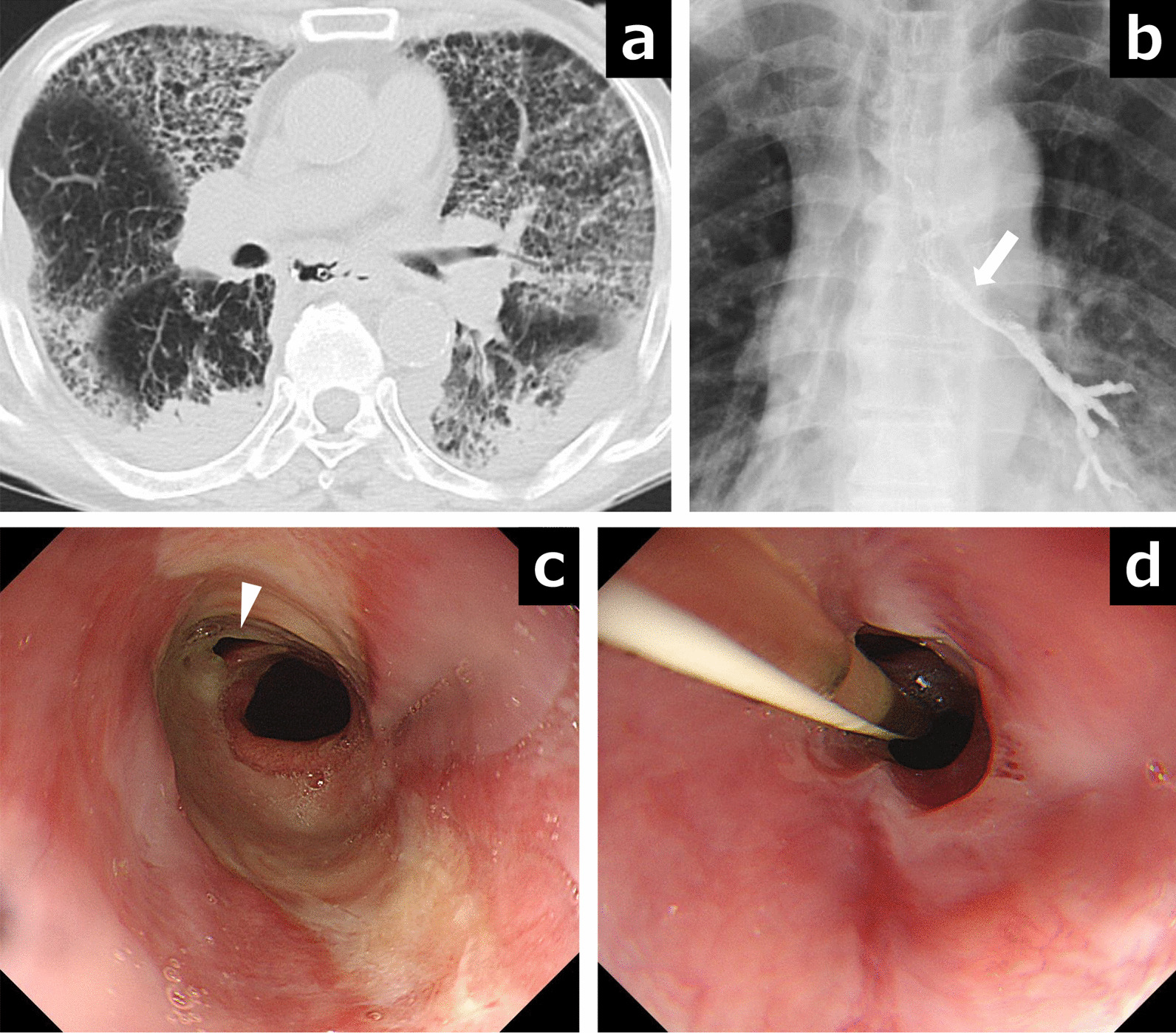
The findings of esophago-left bronchial fistula are shown. **a** CT images showed bilateral severe pneumonia. **b** Gastrografin contrast study showed leakage into the left main bronchus (white arrow). **c** A fistula (white arrowhead) was detected in the anterior wall of the esophagogastric anastomotic area by upper endoscopy. d The esophageal ulcer shrank, but the fistula did not close after 1 month

Four months after surgery, he was re-admitted due to dyspnea, chronic cough, fever, and dysphagia. A blood gas analysis on admission reveled respiratory failure with a partial pressure of arterial oxygen of 59.2 mmHg and a partial pressure of arterial carbon dioxide of 36.8 mmHg (O_2_, 6 L/min), and CT images revealed severe bilateral pneumonia and pneumothorax (Fig. 2a). A Gastrografin contrast study showed a persistent fistulous tract between the gastric tube and the left main bronchus (Fig. 2b). Upper endoscopy showed a circumferential ulcer of the esophagus proximal to the anastomosis and a fistula in the anterior wall of the distal esophagus (Fig. 2c). After these examinations, a nasogastric drainage tube and nutritional feeding tube from the nose to the duodenum were inserted, and treatment against pneumonia and the esophageal ulcer using a proton pump inhibitor was performed for 1 month. Although the esophageal ulcer shrank and the EBF had become slightly smaller after this treatment (Fig. 2d), digestive fluid and sputum frequently refluxed into the lung, and the respiratory disorder remained.

We considered that right thoracotomy would be too invasive and dangerous for patient who developed extensive right pyothorax after esophagectomy. We also considered separating the respiratory and gastrointestinal tracts by constructing an esophagostomy, but this method could cause severe mediastinitis due to the loss of blood flow in the esophagus after esophagectomy. Therefore, we decided to perform a three-step operation for this EBF.

**Fig. 3:**
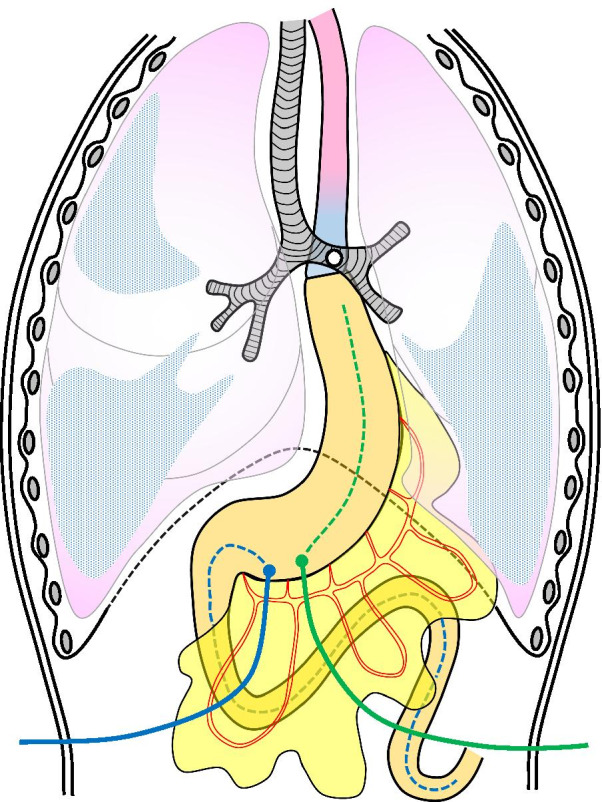
Schema of the first procedure of the three-step operation. A decompression catheter (green line and dotted line) and a feeding tube (blue line and dotted line) were inserted via gastrostomies in the gastric tube

The first procedure was aimed at maintaining stable tube nutrition and continuous drainage of digestive fluid in the gastric tube to improve his pneumonia. We inserted a decompression catheter into the gastric tube as a gastrostomy and a feeding tube to the jejunum from the gastric tube by laparotomy under general anesthesia (Fig. 3). This operation enabled stable nutritional management and treatment of pneumonia, and although there were two tubes in the abdomen, the nasogastric tubes could be removed, allowing the patient to be discharged temporarily. However, over the next 9 month, he repeatedly developed aspiration pneumonia.

**Fig. 4:**
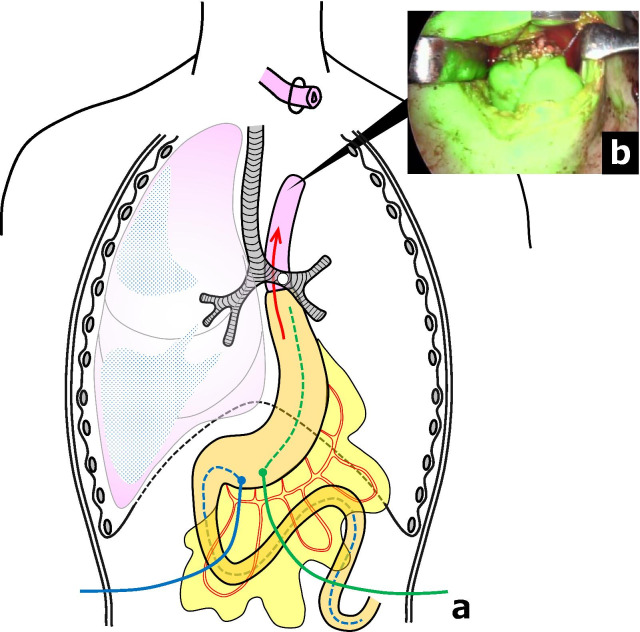
Schema of the second procedure of the three-step operation. **a** The cervical esophagus was brought to the left cervical region as an esophagostomy. **b** Inflow of ICG is apparent from the gastric tube through the esophagogastric anastomosis

The second procedure was performed 14 months after the esophagectomy to bring the cervical esophagus to the left cervical region as an esophagostomy (Fig. 4a). We performed intraoperative ICG-guided evaluation of blood flow through the anastomosis in the hope of confirming blood supply by collateral circulation through the anastomotic line from the gastric tube. The esophagus was taped at the left neck, the planned esophagostomy site was clamped, and ICG 25 mg/5 mL was gently injected via the peripheral blood route. The ICG fluorescence was detected with near-infrared light visualization (Stryker Co., Kalamazoo, USA). Within about 30 s after the ICG solution was injected, blood flow in distal side of the esophagus was confirmed from the gastric tube through the esophagogastric anastomosis (Fig. 4b). This procedure prevented dripping of oral saliva and digestive fluid and eliminated direct inhalation of digestive fluid via the esophago-bronchial fistula, following which the patient then underwent 3 months of physical rehabilitation and nutritional support to prepare for reversal of the esophageal discontinuity.

**Fig. 5:**
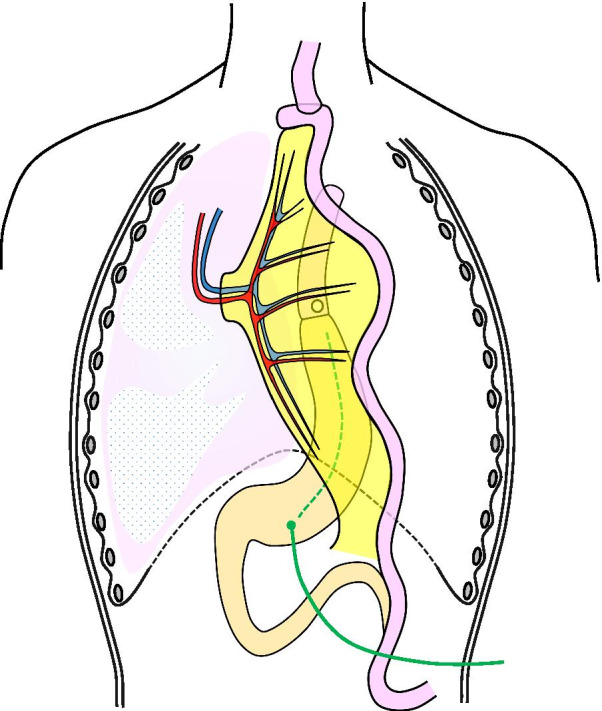
Schema of the final procedure of the three-step operation. The esophagus was reconstructed using a pedicle jejunum in a Roux-en-Y anastomosis via a subcutaneous route with microvascular anastomosis of the right internal thoracic vessels and the second jejunal vessels

The third procedure was performed 17 months after the esophagectomy. The esophagus was reconstructed using a pedicle jejunum in a Roux-en-Y anastomosis via a subcutaneous route. After confirming sufficient blood flow, the second and third branches of the jejunal artery and vein were ligated, and a long segment of the jejunum was pulled up subcutaneously. Microvascular anastomoses of the right internal thoracic arteriovenous and second jejunal arteriovenous vessels were performed by plastic surgeons. The esophagostomy was closed, the cervical esophagus and the lifted jejunum were manually anastomosed, and the end of the pedicled jejunum was anastomosed to the jejunum in a Roux-en-Y configuration (Fig. 5). Oral intake was started on POD 7, and a barium swallow study showed smooth oral intake. The patient was discharged on POD 31.

## Discussion and conclusions

Most of case series on EBF after esophagestomy are from late 1990s onward, and it is a very rare complication with an incidence of 0.26–3.15% [4–15]. EBF is one of the most difficult complications to deal with after esophagectomy and reconstruction via a gastric tube thorough the posterior mediastinal route. Development of EBF compromises the integrity of both the airway and the digestive tract, resulting in respiratory failure due to severe aspiration pneumonia, and even with surgery, postoperative mortality is very high (Table 1).

**Table 1:** Comparison of recent reports describing the management of fistula between the airway and reconstructed conduit after esophagectomy

Autor [reference]	Data	Patients with fistula	Incidence (%)	Operative mortality (%)
Bartels et al. [4]	1998	31	0.51	n/a
Buskens et al. [5]	2001	6	0.26	0/4 (0)
Mangi et al. [6]	2002	2	n/a	0/2 (0)
Shen et al. [7]	2010	11	n/a	2/35 (5.7*)
Yasuda et al. [8]	2012	10	1.65	3/6 (30)
Schweigert et al. [9]	2012	7	3.15	2/2 (100)
Kuwabara et al. [10]	2013	9	1.89	1/4 (25)
Wang et al. [11]	2014	7	0.64	2/3 (66.8)
Morita et al. [12]	2015	6	0.26	0/2 (0)
Lambertz et al. [13]	2016	13	1.08	4/7 (57.1)
Balakrishnan et al. [14]	2018	3	0.28	n/a
Changchu et al. [15]	2020	26	0.41	11/26 (42.3)

*n/a* not available

*Mortality rate regarding the total number of 35 patients with fistula

Various causes of EBF such as anastomotic leakage, gastric tube necrosis, ulcer of the stomach, and bronchial ischemia have been reported. We consider the direct cause of the EBF in our case to be ischemia occurring at the distal end of the esophagus and severe reflux esophagitis. The patient was obese and had a thick neck, and the esophagus remained too long due to the patient’s physique when the esophagus and gastric tube were manually anastomosed in the neck. Furthermore, because we had dissected the esophageal branches of the inferior thyroid artery and tracheoesophageal arteries, which supply blood to the cervical esophagus during upper mediastinum dissection [16], we speculated that poor blood flow to the anastomotic site, which reached the level of the tracheal bifurcation, combined with severe reflux esophagitis caused by the intrathoracic anastomosis, may have resulted in ulceration and fistula formation.

Surgical treatment generally consists of a direct approach with dissection of the fistula and closure of the bronchial and gastric tube defects, but there is no established surgical procedure. Shen et al. [7] described that single-stage primary repair of tracheoesophageal fistula with tissue flap interposition can safely be performed successfully in the majority of patient. On the other hand, some reports classify fistula according to type and location, and suggest that the indications for surgery should be carefully determined [10, 15]. The interposed vital tissue used for repair are similarly varied, with latissimus dorsi muscle flap, major pectoral muscle flap, pericardial flap and pedicled muscle flap being reported [12, 17].

These repairs of the EBF usually require right thoracotomy and left one-lung ventilation. However, when an esophageal left bronchial fistula is formed, as in our case, there are two important problems.

The first is that the anesthesia for left one-lung ventilation required for right thoracotomy is highly difficult due to the location of the fistula and preoperative pneumonia. Moreover, for patients who are in severe respiratory failure preoperatively due to EBF, general anesthesia itself is very invasive and risky. Second, even if an esophagostomy is performed to separating the respiratory and gastrointestinal tracts to control the aspiration pneumonia, residual esophagus, including the EBF that has lost blood flow remains in the mediastinum. Initially we considered a two-step operation for separate the respiratory and gastrointestinal tracts by constructing an esophagostomy at the left cervical region without thoracotomy to prevent dripping of oral saliva. However, we decided that it would be impossible to dissect the esophagus to the tracheal bifurcation and pull it up to the neck using only the cervical approach. Therefore, we selected a safe surgical approach without thoracotomy that is systematically divided into three-step.

Several Successful cases of multi-step operation have been reported as a surgical strategy for patients who have difficulty with thoracotomy, which was partially similar to our treatment concept [18, 19]. Bamba et al. [18] successfully performed a three-step operation for esophagorespiratory fistula following airway stenting for stenosis of tracheal anastomosis, but blood flow to the remaining esophagus in the mediastinum was maintained via the proper esophageal artery, which was different from our case. Ibuki et al. [19] performed a two-step operation for EBF after esophagectomy, but unlike our case, they were able to dissect the entire esophagus using only the cervical approach.

There are several case reports of fully developed neovascularization from the gastric tube to the anastomosis, which is an important finding to ensure blood flow in the residual esophagus, the second problem [20–22]. Hara et al. [20] reported a successful case of distal part resection of gastric duct cancer after esophagectomy by blood flow evaluation and stated that the interval from esophagectomy to neovascularization was a minimum of 10 months and a maximum of 154 months, but less than one year is very rare. For this reason, we decided to allow 14 months for esophagostomy. For this reason, we decided to allow 14 months for esophagostomy.

In the three-step operation we performed, the first procedure improved the pneumonia by maintaining tube nutrition and draining digestive fluid in the gastric tube. This also allowed time for neovascularization to develop from the wall of the gastric tube through the anastomosis. In the second procedure, ICG-guided blood flow evaluation of the distal side of the esophagus enabled prevention of oral saliva dripping and upstream regurgitation from the stomach through creation of the esophagostomy. Finally, after the patient underwent physical rehabilitation and nutritional management, the third procedure was performed to restore esophageal continuity using an esophago-jejunal bypass.

If a patient’s general condition is good, we may perform the second and third procedure at the same time. In recent years, successful cases of endoscopic fistula closure have been reported [23, 24], and this may become a treatment option. However, we did not choose endoscopic treatment in our case because we were concerned that aspiration pneumonia and respiratory failure due to the EBF would be exacerbated by endoscopic insufflation.

This three-step operation resulted in a favorable outcome for our patient with EBF after esophagectomy. This approach can be safe and useful in patients with respiratory failure due to EBF, especially those in whom right thoracotomy with left one-lung ventilation would be difficult to perform.

## Data Availability

Not applicable.

## Abbreviations

CT: Computed tomography
EBF: Esophago-bronchial fistula
ICG: Indocyanine green
POD: Postoperative day
